# Females tend to prefer genetically similar mates in an island population of house sparrows

**DOI:** 10.1186/1471-2148-14-47

**Published:** 2014-03-12

**Authors:** Coraline Bichet, Dustin J Penn, Yoshan Moodley, Luc Dunoyer, Elise Cellier-Holzem, Marie Belvalette, Arnaud Grégoire, Stéphane Garnier, Gabriele Sorci

**Affiliations:** 1 Biogéosciences, UMR CNRS 6282, Université de Bourgogne, 6 Boulevard Gabriel, 21000 Dijon, France; 2 Konrad Lorenz Institute of Ethology, Department of Integrative Biology and Evolution, University of Veterinary Medicine, Savoyenstr 1a, Vienna A-1160, Vienna, Austria; 3 CEFE, UMR-CNRS 5175, Université de Montpellier 2, 1919 route de Mende, 34293 Montpellier, France

**Keywords:** Sexual selection, Mate choice, *Passer domesticus*, Major Histocompatibility Complex (MHC), Microsatellites, Extra-pair paternity

## Abstract

**Background:**

It is often proposed that females should select genetically dissimilar mates to maximize offspring genetic diversity and avoid inbreeding. Several recent studies have provided mixed evidence, however, and in some instances females seem to prefer genetically similar males. A preference for genetically similar mates can be adaptive if outbreeding depression is more harmful than inbreeding depression or if females gain inclusive fitness benefits by mating with close kin. Here, we investigated genetic compatibility and mating patterns in an insular population of house sparrow (*Passer domesticus*), over a three-year period, using 12 microsatellite markers and one major histocompability complex (MHC) class I gene. Given the small population size and the distance from the mainland, we expected a reduced gene flow in this insular population and we predicted that females would show mating preferences for genetically dissimilar mates.

**Results:**

Contrary to our expectation, we found that offspring were less genetically diverse (multi-locus heterozygosity) than expected under a random mating, suggesting that females tended to mate with genetically similar males. We found high levels of extra-pair paternity, and offspring sired by extra-pair males had a better fledging success than those sired by the social male. Again, unexpectedly, females tended to be more closely related to extra-pair mates than to their social mates. Our results did not depend on the type of genetic marker used, since microsatellites and MHC genes provided similar results, and we found only little evidence for MHC-dependent mating patterns.

**Conclusions:**

These results are in agreement with the idea that mating with genetically similar mates can either avoid the disruption of co-adapted genes or confer a benefit in terms of kin selection.

## Background

The adaptive function of mate choice remains unclear and one of the most challenging problems in behavioural ecology [1-3]. Choosy females can potentially obtain direct or indirect genetic benefits for their progeny (genetic quality or compatibility) [3-5]. Females that avoid mating with related or genetically similar mates avoid the costs of inbreeding depression [6-10]. Inbreeding avoidance may also increase the genetic diversity of progeny, which might confer fitness benefits in temporally and spatially heterogeneous environments. Yet, several recent studies have surprisingly reported evidence for mate choice for *genetically related* reproductive partners [11-13]. It is unclear whether or how inbreeding *per se* could be beneficial, though such findings may be due to outbreeding avoidance. If local environmental conditions selectively favour co-adapted ensembles of genes, mating with genetically distant partners could disrupt these assemblages and result in a loss of fitness (outbreeding depression) [14-17]. Bateson [18,19] suggested that maximal reproductive success may be achieved by pairs with intermediate genetic relatedness, and there is support for his ‘optimal outbreeding’ hypothesis [20-22]. Mating preferences for kin could also allow females to increase their inclusive fitness [23-25] in the absence of significant inbreeding depression [18,26,27]. Similarly, selecting kin as social mates may improve cooperation between the sexes and reduce sexual conflict over parental investment [28].

The rapid development of molecular genetic tools in recent years has considerably aided mate choice research in several ways. First, although the vast majority of bird species are socially monogamous, many species also engage in extra-pair copulations so that broods are usually composed of chicks sired by different fathers [29-32]. Genetic paternity analyses allow the detection of extra-pair (EP) paternity and identification of EP males and EP offspring. Moreover, extra-pair mating presents an opportunity to examine the processes governing mate choice in the absence of any potential direct benefit since the extra-pair male does not contribute to parental care. According to the inbreeding avoidance hypothesis, for example, females mated with closely related males should engage in extra-pair copulations with genetically dissimilar mates [3], whereas the outbreeding avoidance and the kin selection hypotheses predict that females should engage in extra-pair copulations with genetically similar males [8,33]. Second, molecular genetic tools make it possible to infer individuals’ genome-wide diversity and relatedness between partners using both neutral loci (e.g. microsatellites) and functional selected genes [1-5]. Third, molecular tools have helped to test the hypothesis that genetic benefits from mate choice include increasing offspring heterozygosity at the major histocompatibility complex (MHC) loci [34-39]. MHC genes encode cell-surface glycoproteins that control antigen presentation, and MHC heterozygotes are supposed to better face infectious diseases [40,41]. Yet, just as different species show inbreeding or outbreeding preferences, recent work indicates the MHC-dependent mating preferences can be disassortative or assortative for alleles, disassortative for allelic diversity, or for specific alleles. It has been suggested that ‘if there is an optimal level of MHC heterozygosity for combating infections, then females should prefer to mate with males that have intermediate levels of MHC dissimilarity’ [35], and subsequent work has provide evidence that female preferences depend on males’ individual allelic diversity [39,42,43].

We studied mate choice both for social and extra-pair mates in a small insular population of the house sparrow (*Passer domesticus*), using microsatellite loci and MHC class I genes, over three consecutive years. Mating patterns were assessed by examining offspring genetic diversity. This indirect assessment only allowed us to infer realized mate choice, whereas assessing female preference would have required letting females choose a partner in the absence of constraints [44]. Given the small population size and the isolated nature of the studied population, we expected reduced gene flow. This led us to predict that females should preferentially mate with diverse and genetically dissimilar males to i) reduce the risk of inbreeding; ii) enhance genetic diversity of their progeny.

## Methods

### The study population

The house sparrow population studied here is located at Hoëdic, a small (2.08 km^2^) island off the French coast of Brittany (47°20’24.40”N-2°52’43.09”W). Adult house sparrows were captured using mist nets and banded with a metal ring and a unique combination of coloured rings which allowed individual recognition. At the first capture, we obtained a small amount of blood (20 μl) by brachial vein puncture and stored it in 500 μl of Queen’s Lysis Buffer (QLB) [45]. We monitored pairs breeding in nest boxes that were set up in the village, from 2009 to 2011. For logistic reasons, we were only able to monitor the first two broods during each year, even though house sparrows can lay up to 3-4 clutches per breeding season. Between late April and the end of June, we visited nest boxes at least twice per week and recorded clutch size and the number of hatched and fledged chicks. When chicks were 8 days old, they were banded with a metal ring and a drop of blood was collected and stored as for adults. The identities of social parents were assessed during focal observations when adults were brooding or feeding the chicks. Sample sizes are summarized in Table 1. Ringing licence and permit to take blood samples were given by the Muséum National d’Histoire Naturelle (Paris) and the Préfecture du Morbihan.

**Table 1:** Total number of individuals, breeding pairs, and individuals genotyped

**Year**	**Number of sampled individuals**	**Number of breeding pairs with known identity**	**Number of microsatellite genotyped individuals (number of chicks)**	**Number of MHC genotyped individuals (number of chicks)**
2009	225	15	222 (51)	190 (51)
2010	341	40	335 (82)	294 (82)
2011	316	49	311 (89)	275 (89)
Total*	574	96	565 (222)	494 (222)

*Total of unique individuals.

We estimated population size using the POPAN module of the software MARK [46]. POPAN gives an estimate of the population size while taking into account the probabilities of recapture (p) and survival (ϕ), as well as the probability of new individuals entering the population (p_ent_). We ran models where each of the three parameters was either constant or varied as a function of capture session (time) (three sessions per year, three years = nine capture sessions). The best model was selected based on the AIC criterion and was the model where the three parameters varied with time. We also used the U-CARE module to check any violation of the assumptions underlying the use of capture-mark-recapture models and did not find any departure from these assumptions [47].

### Microsatellite genotyping

DNA was extracted using the Wizard® SV 96 Genomic DNA Purification kit (Promega) according to the manufacturer’s instructions. All individuals were genotyped using the following twelve microsatellite loci: PdomD09, PdomA08, PdomB01, PdomH05 [48], Mjg1 [49], Ase18 [50], Pdo3, Pdo5 [51], Pdo1 [52], Pdo10 [53], Pdo16 and Pdo27 [54]. Polymerase chain reactions (PCRs) were performed in a final volume of 10 μl including 10 to 50 ng of DNA, 2 μl of 5X buffer, 1.5 to 2 mM of MgCl_2_, 400 μl of dNTPs, 1 μM of each primers and 0,2 U of *Taq* DNA polymerase (Promega). The PCR program comprised: 94°C 3 to 4 min, 30 to 35 cycles of 94°C 20s, 20s for annealing (48°C to 62°C according to the different loci), and 72°C 30 to 40s, followed by a final extension of 72°C 5 to 7 min. Samples were then run in an ABI3730 automated sequencer. Allele sizes were determinated using GeneMapper v4.0.

### MHC class I genotyping

We amplified the MHC class I exon 3, which corresponds to the highly variable peptide-binding region (PBR) of the protein [55]. Passerines have been shown to have several loci at the MHC class I exon 3 due to gene duplication and fragmentation, which makes it impossible to determine the number of amplified loci or estimate heterozygosity at each locus [42,55-58].

PCR amplifications were performed using a fluorescent (6’FAM) labelled primer (A23M – GCG CTC CAG CTC CTT CTG CCC ATA) and an unlabeled primer (A21M – GTA CAG CGC CTT GTT GGC TGT GA). PCRs were performed in a final volume of 10 μl, including 50 to 100 ng of genomic DNA, 0.6 μM of each primer and 5 μl of Multiplex PCR reagent (QIAGEN GmbH) containing hot-start DNA polymerase, buffer and dNTPs. The PCR program began with 5min initial denaturation at 95°C, followed by 35 cycles of 30s denaturation at 94°C, 90s annealing at 56°C and 90s extension at 72°C. A final elongation step was run for 10 min at 72°C. To control for PCR artefacts, we used 2 negative controls, for PCR and for sequencer, by adding purified water instead of DNA or PCR products. MHC diversity was screened using capillary electrophoresis single conformation polymorphism (CE-SSCP) [43]. PCR samples were prepared for electrophoresis by combining 1 μl PCR product, with 8.75 μl Hi-Di formamide and 0.25 μl of in-house prepared ROX size standard [59]. This mix was heated for 5min at 95°C to separate the complementary DNA strands. Electrophoresis was conducted in an automated DNA sequencer (ABI PRISM 3130 xl automated DNA Sequencer, Applied Biosystems). The retention time of allelic variants was assessed relative to the ROX size standard.

### Statistical analyses

#### Estimations of within-individual genetic diversity and between-individual dissimilarity

Genome-wide inbreeding was assessed with individual Internal Relatedness (IR, [60]). IR corresponds to the number of homozygous loci, divided by the number of genotyped loci, weighted by the allele frequencies. To measure the genetic similarity between paired males and females, we also computed unbiased pairwise relatedness (r, [61]), where each locus is weighted using the method described in [62,63]. IR and r were assessed from multilocus microsatellite genotypes.

Allele-sharing was calculated to estimate MHC similarity between males and females forming a pair-bond. Allele-sharing is twice the number of shared alleles divided by the number of different alleles of each individual [D = 2F_ab_/(F_a_ + F_b_)] [42,64].

### Paternity analysis

In each nest, social parents were identified during the brooding and chick feeding period. To assess extra-pair paternity, we used the likelihood-based approach implemented in the software CERVUS 3.0 [65]. The software allows excluding and assigning putative fathers based on their multi locus genotypes. The probability of exclusion and assignment was fixed to 95%. We also tested if there was any mismatch between the maternal identity based on the field observations and the one based on microsatellites. Maternal mismatches would indicate that brood parasitism had occurred. Over the entire study period, in only two instances we found a mismatch between the maternal identity based on the field observations and the genetic markers. However, these mismatches involved complete clutches which likely reflect errors in the reading of the color bands rather than brood parasitism. Accordingly these two records were excluded from the statistical analyses. We considered a chick as being extra-pair if it was sired by a male other than the social male that was identified by field observations. We also tested whether there was any evidence suggesting a departure from Hardy-Weinberg equilibrium of the microsatellite loci using CERVUS 3.0 [65]. We did not find any departure from Hardy-Weinberg equilibrium (all p < 0.05; Table 2).

**Table 2:** Number of alleles (k), number of genotyped adults (N), observed heterozygosity (Hobs), expected heterozygosity (Hexp) and deviation from Hardy-Weinberg equilibrium (HW) for each microsatellite locus over the three study years (NS = non significant)

**Locus**	**k**	**N**	**Hobs**	**Hexp**	**HW**
PdomD09	8	343	0.73	0.76	NS
PdomA08	12	335	0.72	0.80	NS
PdomB01	9	343	0.62	0.61	NS
PdomH05	16	343	0.70	0.71	NS
Mjg1	21	343	0.90	0.92	NS
Ase18	15	343	0.86	0.88	NS
Pdo3	16	343	0.91	0.89	NS
Pdo5	18	343	0.83	0.84	NS
Pdo1	15	334	0.84	0.86	NS
Pdo10	13	337	0.87	0.88	NS
Pdo16	13	343	0.81	0.82	NS
Pdo27	14	322	0.84	0.84	NS

### Mate choice

The null hypothesis of random mating with respect to parental relatedness was tested by comparing observed chick IR values to expected distributions under this null hypothesis. We computed observed IRs for i) the overall sample of chicks produced over the three-year study period (n = 222), ii) chicks produced by social males (n = 111), and iii) chicks produced by extra-pair males (n = 65). Over the entire sample of 222 chicks, paternal identity could not be assigned for 46 chicks. We also compared the relatedness (r) between pair members (between the female and her social mate, and her genetic mate when she engaged in EP copulations) to the expected distribution of values under random mating. Expected values were generated using the software STORM [66] by randomly sampling (1000 iterations for each year) reproductive males and females that were observed in a given year in order to generate the same number of chicks and mating pairs to the observed ones in the same year.

MHC allele-sharing (D, between the female and her social mate, and her genetic mate when she engaged in EP copulations) was compared with the distribution of expected D values obtained by randomly generating mating pairs (1000 bootstraps for each year, R version 2.15.0, R Development Core Team 2011). As for IR and r, D was computed using social and extra-pair partners.

### Hypothesis testing

We used General Linear Mixed Models (GLMMs) to test the hypothesis that females mated with more closely related social males would also engage in extra-pair fertilization. Brood type was modeled as a binary response variable (with or without extra-pair chicks). The explanatory (fixed) variables were social male IR, relatedness to the female (r), band-sharing within the male-female pair, and year. Since some females laid several clutches during the three-year period covered by the study, female identity nested within year was set as a random factor.

We also compared the hatching and fledging success of broods with no extra-pair chicks to the hatching and fledging success of broods containing at least one extra-pair young. Here, fledging success was entered as a binomial response variable (fledged or not), year and brood type (broods with extra-pair or no extra-pair young) were also included as fixed factors and female identity was nested within year as a random factor.

Internal Relatedness (IR) of chicks produced by social males was compared to IR of chicks produced by extra-pair males using a GLMM with a binomial distribution of errors. Year, chick type (sired by the social or the extra-pair male), and their interactions were added as fixed factors. Female identity was nested within year and entered as a random factor.

We also compared IR, r and D between social and extra-pair males, for the restricted sample of females that engaged in extra-pair copulations, with the aim of investigating if male genetic characteristics and the relatedness with the female affected the likelihood of being a social or an extra-pair male. We constructed a GLMM where male status (social or extra-pair) was entered as a binomial response variable. Male IR, D, r and year were included as fixed factors. Breeding event nested within female and within year was also declared as a random factor. When a female mated with several extra-pair mates during a single reproductive event, we computed the mean IR, r and D and used these values in the statistical models.

We used a similar GLMM to test if within-brood chicks sired by the social or the extra-pair male(s) differed in their fledging success. Fledging success corresponds to the number of fledged chicks sired by a given male, divided by the total number of eggs laid and this was modeled as a binomial response variable. Male type (social or extra-pair), year and their interaction were added as fixed factors. Breeding event, nested within female identity and year was added as a random factor.

We used the package lme4 [67], implemented in R 2.15.0 to run all GLMMs. We used the information-theoretic (IT) approach to perform model selection [68]. Model support was assessed using the corrected version of Akaike Information Criterion (AICc) for small sample sizes, and ΔAIC was used to infer support for models in the candidate set [69]. ΔAIC corresponds to the difference in AICc of the focus model minus the AICc of the best model (the model with the lowest AIC) [68]. We calculated the Akaike weights (*ω*) for each model, which is the probability that a model is selected as the best in a model set [68]. Using the package MuMIn [70], we also calculated the summed AIC weight (ΣAIC*ω*) for each variable. This corresponds to the sum of the weights of the models in which the variable is present and can be interpreted as the probability that a given variable is retained in the selected model [68,71]. Following Burnham and Anderson [68], we considered that a model had substantial empirical support if its ΔAIC was lower than 2.

## Results and discussion

The number of alleles for each microsatellite locus varied between 8 and 21, whereas observed heterozygosity varied between 0.70 and 0.91 (Table 2). Individuals had a mean of 21.50 (± 1.05 SE) microsatellite alleles over 12 loci, and a mean of 2.31 (± 0.05 SE) MHC class I alleles. The individual MHC allele number varied between 1 and 6 (from as many as 3 class I loci amplified) and a total of 37 MHC alleles were found in the entire population.

Over the three study years the average population size of adult birds was 204 individuals (± 21 SE). Therefore, we sampled a substantial fraction of the total estimated breeding population each year.

In 2009, 2010 and 2011, respectively 35.3, 40.2 and 48.3% of chicks were from extra-pair matings. Similarly, 64.3, 57.7 and 69.0% of broods contained at least one extra-pair young, respectively.

**Figure 1:**
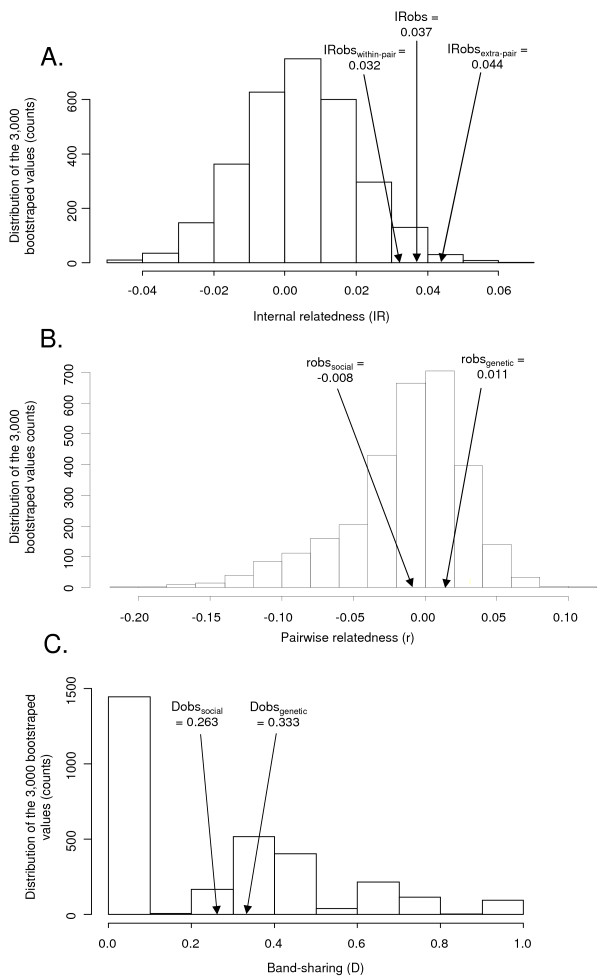
**Distribution of the 1,000 bootstraped Internal Relatedness IR (A), pairwise-relatedness r (B) and MHC allele-sharing (C).** IRobs_,_ IRobs_within-pair_ and IRobs_extra-pair_ correspond to the observed IR computed on the entire sample of chicks, chicks produced by social males and chicks produced by extra-pair males, respectively. robs_social_, Dobs_social_ correspond to the observed r and allele-sharing between the female and her social mate. robs_genetic_, Dobs_genetic_ corresponds to the observed r and allele-sharing between the female and her extra-pair mate.

The observed IR computed over the entire sample of chicks (IRobs) was significantly higher than expected under random mate choice (mean = 0.037 ± 0.001 SE; n = 222; p = 0.020; Figure 1A). The results were similar when IR was computed on within-pair only (IRobs_within-pair_ = 0.032 ± 0.014 SE; p = 0.046; n = 112), or extra-pair only chicks (IRobs_extra-pair_ = 0.044 ± 0.020 SE; p = 0.009; n = 53) (Figure 1A).

Mean pairwise relatedness (robs_social_) between females and their social mates was **-**0.008 (± 0.016 SE; n = 96) and did not differ from expected values under a random mate choice (Figure 1B). Mean pairwise relatedness (robs_genetic_) between females and their genetic mates was 0.011 (± 0.018 SE; n = 79) and did not differ from expected values under random mate choice (Figure 1B).

MHC allele-sharing (Dobs_social_) between females and their social mates and allele-sharing (Dobs_genetic_) between females and their extra-pair mates where respectively 0.263 (± 0.024 SE; n = 56) and 0.333 (± 0.045 SE; n = 75), and did not differ from expected values under random mate choice (Figure 1C).

None of the measures of relatedness between the female and her social mate (r), and inbreeding level of the male (IR) affected the female likelihood to engage in extra-pair fertilizations, since the model with the lowest AIC value was the null model (Table 3, n = 70). Similarly, none of the variables improved the fit of the model exploring the variation in hatching success (the null model had the lowest AIC and the highest *ω* (Table 4, n = 61). However, fledging success did vary among years (0.89 in 2009, 0.75 in 2010, and 0.59 in 2011) and the model including year had the lowest AIC and the highest *ω* values (Table 4, n = 53). IR of chicks was not influenced by mating type (social or extra-pair) but tended to vary across years. However, we should note that the null model is very close suggesting that the effect of year is rather weak (Table 5, n = 222).

**Table 3:** **A: GLMM exploring the effects of Internal Relatedness (IR), pairwise relatedness (r), and MHC allele-sharing (D) of the social male on the female likelihood to engage in extra-pair fertilizations, B: Relative variable importance given by Akaike weights (ΣAIC *ω *)**

**A.**	**Variables**	**Model**	**K**	**AICc**	**ΔAICc**
	Brood type (broods with extra-pair chicks vs. broods with no extra-pair chicks)	Null	1	90.6	0.00
	(*n* = 66)	D	2	91.4	0.78
		IR	2	92.0	1.37
		r	2	92.0	1.39
		D + r	3	92.5	1.88
		D + IR	3	93.1	2.53
		IR + r	3	93.4	2.86
		Year	3	94.3	3.74
		D + IR + r	4	94.4	3.8
		D + year	4	95.5	4.89
		IR + year	4	95.6	5.02
		r + year	4	95.8	5.18
		D + r + year	5	96.7	6.08
		IR + r + year	5	97.2	6.61
		D + IR + year	5	97.2	6.62
		D + IR + r + year	6	98.6	8.03
**B.**	**Variables**	**Source of variation**	**ΣAIC** ***ω***		
	Brood type (broods with extra-pair chicks vs. broods with no extra-pair chicks)	D	0.39		
		r	0.34		
		IR	0.31		
		Year	0.13		

K = number of parameters.

**Table 4:** **A: GLMM exploring the effects of brood type (broods with extra-pair chicks vs. broods with no extra-pair chicks) on hatching and fledging success, B: Relative variable importance given by Akaike weights (ΣAIC *ω *)**

**A.**	**Variables**	**Model**	**K**	**AICc**	**ΔAICc**	***ω***
	Hatching success	Null	1	74.1	0.00	0.523
	(*n* = 65)	Brood type	2	76.1	1.96	0.196
		Year	3	76.6	2.46	0.153
		Year + brood type + year* brood type	5	78.1	3.97	0.072
		Year + brood type	4	78.6	4.47	0.056
	Fledging success	Year	3	96.6	0.00	0.722
	(*n* = 55)	Year + brood type	4	99.0	2.42	0.215
		Null	1	102.7	6.07	0.035
		Year + brood type + year* brood type	4	104.1	7.54	0.017
		Brood type	2	104.9	8.25	0.012
**B.**	**Variables**	**Source of variation**	**ΣAIC** ***ω***			
	Hatching success	Brood type	0.32			
		Year	0.28			
		Year*brood type	0.07			
	Fledging success	Year	0.95			
		Brood type	0.24			
		Year*brood type	0.02			

K = number of parameters. Asterisk means the interaction.

**Table 5:** **A: GLMM exploring the effects of chick type (sired by the social or extra-pair mate) on chick multi-locus internal relatedness (IR), B: Relative variable importance given by Akaike weights (ΣAIC *ω *)**

**A.**	**Variables**	**Model**	**K**	**AICc**	**ΔAICc**	***ω***
	IR	Year	3	−231.1	0.00	0.308
	(*n* = 222)	Null	1	−231.0	0.05	0.300
		Year + chick type + year*chick type	5	−229.5	1.55	0.142
		Year + chick type	4	−229.4	1.70	0.132
		Chick type	2	−229.2	1.90	0.119
**B.**	**Variables**	**Source of variation**	**ΣAIC** ***ω***			
	IR	Year	0.58			
		Chick type	0.39			
		Year*chick type	0.14			

K = number of parameters. Asterisk means the interaction.

We then focused on broods that contained both within and extra-pair chicks to compare the genetic characteristics (IR, r and D) between social and extra-pair mates. The best model was the one that included pairwise relatedness (r, Table 6, n = 60). The ΔAICc and ΣAICω of the other competitive models also suggested a possible role for MHC band sharing and pairwise relatedness (Table 6). The mean pairwise relatedness was 0.074 (± 0.029 SE, n = 30) between females and extra-pair mates whereas it was 0.008 (± 0.029 SE, n = 30) between females and social mates. MHC allele-sharing was 0.34 (± 0.050 SE, n = 30) between females and extra-pair mates, and 0.24 (± 0.047 SE, n = 30) between females and social mates. We should however mention that the null model is also competitive suggesting that the contribution of these variables is weak.

**Table 6:** **A: GLMM exploring the effects of Internal Relatedness (IR), pairwise relatedness to the female (r), and MHC allele-sharing (D) on the likelihood of being a social or an extra-pair male, B: Relative variable importance given by Akaike weights (ΣAIC *ω *)**

**A.**	**Variables**	**Model**	**K**	**AICc**	**ΔAICc**	***ω***
	Male type (social vs. extra-pair)	r	2	98.1	0.00	0.183
	(*n* = 60)	D	2	98.3	0.21	0.164
		Null	1	98.5	0.32	0.156
		D + r	3	99.1	0.93	0.115
		IR	2	99.5	1.39	0.091
		IR + r	3	99.8	1.64	0.080
		IR + D	3	100.0	1.83	0.073
		IR + D + r	4	101.0	2.87	0.044
		r + year	4	102.6	4.51	0.019
		D + year	4	102.8	4.65	0.018
		Year	3	102.9	4.77	0.017
		D + r + year	5	103.6	5.43	0.012
		IR + year	4	104.1	5.97	0.009
		IR + r + year	5	104.4	6.26	0.008
		IR + D + year	5	104.6	6.43	0.007
		IR + D + r + year	6	105.6	7.51	0.004
**B.**	**Variables**	**Source of variation**	**ΣAIC** ***ω***			
	Male type (social vs. extra-pair)	r	0.46			
		D	0.44			
		IR	0.32			
		Year	0.09			

K = number of parameters.

Fledging success of chicks sired by extra-pair males (0.41 ± 0.047 SE, n = 30) was higher than for chicks sired by the social mate (0.20 ± 0.044 SE, n = 30). The model including sire type (social vs. extra-pair) had the lowest AIC (Table 7, n = 60), and sire type had a very high ΣAICω (0.99).

**Table 7:** A: GLMM exploring the effects of male type (social vs. extra-pair) on fledging success, Fledging success corresponds to the number of chicks sired by a male, divided by the total number of eggs laid in the clutch, B: Relative variable importance given by Akaike weights (ΣAICω)

**A.**	**Variables**	**Model**	**K**	**AICc**	**ΔAICc**	***ω***
	Fledging success	Male type	2	94.1	0.00	0.891
	(*n* = 74)	Male type + year	4	98.7	4.61	0.089
		Male type + year + year*male type	5	101.8	7.75	0.019
		Null	1	106.9	12.80	0.001
		Year	2	111.3	17.25	0.000
**B.**	**Variables**	**Source of variation**	**ΣAIC** ***ω***			
	Fledging success	Male type	1.00			
		Year	0.11			
		Year*male type	0.02			

K = number of parameters. Asterisk means the interaction.

We expected that the particular demographic and ecological characteristics of the studied insular population of house sparrows would have promoted the evolution of mating preference for dissimilar mates to avoid the depletion of genetic diversity and inbreeding [6-13,72]; however, our results do not provide support for the “inbreeding avoidance” hypothesis. On the contrary, we found evidence suggesting a preference for genetically similar mates. Over the three years covered by the study, we found that offspring were less heterozygous than expected under random mate choice (based on 12 microsatellite loci), though there was little support that MHC class I genes influenced pair formation. This pattern was consistent even when taking into account the relatively high proportion of extra-pair fertilizations. When focusing on broods containing both within and extra-pair chicks, we also found that extra-pair mates tended to be genetically more similar to the females than social mates both for microsatellite markers and MHC genes, even though the statistical support for these findings was less clear-cut than for offspring heterozygosity. Interestingly, in broods containing both within- and extra-pair young, fledging success of chicks sired by extra-pair males was higher than for chicks sired by social mates.

We could only assess realized mate choice (actual mating patterns) and not female preference (the preference that females might express in the absence of constraints). A number of environmental constraints might prevent females from expressing their actual preferences. For example, imperfect sampling of potential mates or the cost of mate searching can indeed affect the pattern of realized mate choice [44].

Our prediction that females would tend to mate with genetically dissimilar mates was based on the assumption that this insular population has low genetic diversity and that it is therefore vulnerable to inbreeding depression. However, as a part of a larger study on the population genetics of the house sparrow, we found that the within-population diversity of both microsatellites and MHC was similar between this (Hoëdic) and six mainland populations located within a radius of 200 km (Bichet et al., unpublished observations). The Hoëdic population was nevertheless genetically differentiated (based on Fst values) from the other populations used in this study. Therefore, while genetic variation has been maintained in this insular population, isolation and reduced gene flow have still produced genetic divergence from the mainland populations (Bichet et al., unpublished observations).

Insofar as the elevated IR of both intra- and extra-pair offspring reflects a female mating preference for genetically similar males (but see findings on pairwise relatedness between mates, Figure 1B), there are several possible mechanisms that could explain such a preference. First, spatially variable environmental conditions (such as variable risks to contract infectious diseases) might have promoted the evolution of co-adapted genes conferring a benefit under the locally prevailing conditions. Second, preference for genetically similar mates might also evolve through kin selection where females seek to increase their own inclusive fitness [23,25]. Our finding that females engaging in extra-pair matings showed a preference for more genetically similar males is also in agreement with the results of two recent avian studies on ground tits (*Parus humilis*) [13] and barn swallows (*Hirundo rustica*) [33]. Wang and Lu [13] showed that even though the propensity of females to engage in extra-pair matings did not depend on the relatedness with the social mates, females nevertheless sought extra-pair copulations with males with whom they were more related to than their social mates. Since there was no cost due to mating with relatives, Wang and Lu [13] suggested that these results support the hypothesis that females gain inclusive fitness by mating with related males.

Findings based on offspring heterozygosity (IR) and based on relatedness between pair members (r) provided a quite different picture, which might appear puzzling. One possible explanation involves the residual variation in IR that is not accounted for by r. Alternatively, we would also like to remind that the two indices refer to two different steps in the process of mate choice, r refers to the similarity between mates, IR refers to the product of the mate choice where recombination, early embryo failure, etc. might contribute to generate the observed discrepancy.

Population structure or ‘environmental constraints’ might also affect pair formation potentially interfering with mating preferences [44]. For instance, related individuals might tend to cluster in the same flock, as reported for instance in lekking peacocks (*Pavo cristatus*) [73] and to occupy nearby nest boxes, which might increase the likelihood of mating with genetically similar mates. However, since the relatedness between social mates was not higher than for random pairs, this type of clustering seems unlikely in the present case.

A large proportion of chicks were sired by an extra-pair mate. The proportion of extra-pair chicks was higher than those previously reported for other house sparrow populations [51,74-77]. One possible explanation is a bias in estimates of extra-pair chicks due to mistaken identification of birds (coloured leg bands), however, this is unlikely as we found a mismatch in maternal identity in only 3% (2/69) of broods (and in both cases the entire brood was assigned to a single mother). In five broods, none of the chicks was assigned to the social father. However, in all of these five cases, chicks were assigned to at least two different males, strongly suggesting that the high proportion of extra-pair chicks was not the result of mistakes in the identification of the social father. Most of the previous work found that extra-pair paternity is low in insular populations [12,51,78], although some exceptions have been reported [79-81], with as much as 55% of extra-pair chicks reported for an insular population of tree swallows (*Tachycineta bicolor*) [75]. The ecological factors that might explain why insular populations have lower or higher levels of extra-pair paternity compared to mainland populations are unclear and contentious. One might speculate that insular populations with high breeding density and synchrony might be more prone to extra-pair copulations because of the increased availability of extra-pair mates, but this would require additional work.

There have been extensive reports of MHC-dependent mating preferences [9,39,82-88]; however, we found no support for a potential role of MHC genes in mate choice, other than the general assessment of relatedness. Insular populations might be less exposed to parasites and pathogens [89-92], which could weaken the selection for specific MHC alleles and diversity (but see [9]).

## Conclusions

These results tend to support the idea that mating with genetically similar mates can either avoid the disruption of co-adapted genes or confer a benefit in terms of kin selection. Definitely, more work should be devoted to the role of MHC-based mate choice in mainland and insular populations that differ in their exposure to infectious diseases.

## Abbreviations

MHC: Major Histocompatibility Complex; EP: Extra-pair; PBR: Peptide-binding region; IR: Internal relatedness; IRobs: Observed internal relatedness; IRobsextra-pair: Observed internal relatedness for extra-pair chicks; IRobswithin-pair: Observed internal relatedness for within-pair chick; MLH: Multi-locus heterozygosity; r: Pairwise relatedness; robssocial: Observed pairwise relatedness for social pairs, between the female and her social male; robsgenetic: Observed pairwise relatedness for genetic pairs, between the female and the male who sired the chicks; D: Number of shared MHC alleles; Dobssocial: Observed number of shared MHC alleles for social pairs; Dobsgenetic: Observed number of shared MHC alleles for genetic pairs; GLMM: General Linear Mixed Model; AIC: Akaike Information Criterion; ω: AIC weights; ∑AICω: Summed AIC weights.

## Competing interests

The authors declare that they have no competing interests.

## Authors’ contributions

Study design: DJP, YM, GS. Data collection in the field: CB, EC-H, MB, SG, GS. Molecular genetic analyses: CB, MB. Statistical analyses: CB, LD. Manuscript writing: CB, DJP, YM, LD, SG, GS. All authors read and approved the final manuscript.

## References

[B1] Andersson MB Sexual Selection 1994 New Jersey: Princeton University Press

[B2] Clutton-Brock T Sexual selection in males and females Science 2007 318 1882 1885 10.1126/science.1133311 18096798

[B3] Kempenaers B Mate choice and genetic quality: a review of the heterozygosity theory Advances in the Study of Behavior, Vol 37 2007 San Diego: Elsevier Academic Press Inc 189 278 Advances in the Study of Behavior

[B4] Neff BD Pitcher TE Genetic quality and sexual selection: an integrated framework for good genes and compatible genes Mol Ecol 2005 14 19 38 15643948 10.1111/j.1365-294X.2004.02395.x

[B5] Mays HL Albrecht T Liu M Hill GE Female choice for genetic complementarity in birds: a review Genetica 2008 134 147 158 10.1007/s10709-007-9219-5 17973192

[B6] Mays HL Hill GE Choosing mates: good genes versus genes that are a good fit Trends Ecol Evol 2004 19 554 559 10.1016/j.tree.2004.07.018 16701321

[B7] Tregenza T Wedell N Genetic compatibility, mate choice and patterns of parentage: invited review Mol Ecol 2000 9 1013 1027 10.1046/j.1365-294x.2000.00964.x 10964221

[B8] Ferretti V Massoni V Bulit F Winkler DW Lovette IJ Heterozygosity and fitness benefits of extrapair mate choice in White-rumped Swallows (Tachycineta leucorrhoa) Behav Ecol 2011 22 1178 1186 10.1093/beheco/arr103

[B9] Richardson DS Komdeur J Burke T von Schantz T MHC-based patterns of social and extra-pair mate choice in the Seychelles warbler Proc Royal Soc B-Biolog Sci 2005 272 759 767 10.1098/rspb.2004.3028 PMC1602051 15870038

[B10] Charlesworth D Willis JH Fundamental concepts in genetics. The genetics of inbreeding depression Nat Rev Genet 2009 10 783 796 10.1038/nrg2664 19834483

[B11] Cohen LB Dearborn DC Great frigatebirds, Fregata minor, choose mates that are genetically similar Anim Behav 2004 68 1229 1236 10.1016/j.anbehav.2003.12.021

[B12] Krokene C Lifjeld JT Variation in the frequency of extra-pair paternity in birds: a comparison of an island and a mainland population of blue tits Behaviour 2000 137 1317 1330 10.1163/156853900501944

[B13] Wang C Lu X Female ground tits prefer relatives as extra-pair partners: driven by kin-selection? Mol Ecol 2011 20 2851 2863 10.1111/j.1365-294X.2011.05070.x 21438933

[B14] Shields WM Thornhill NW The natural and unnatural history of inbreeding and outbreeding The Natural and Unnatural History of Inbreeding and Outbreeding: Theoretical and Empirical Perspectives 1993 Chicago: Chicago University Press 143 169

[B15] Waller NM Thornhill NW The statics and dynamics of mating system evolution The Natural and Unnatural History of Inbreeding and Outbreeding: Theoretical and Empirical Perspectives 1993 Chicago: Chicago University Press 97 117

[B16] Frankham R Ballou JD Eldridge MDB Lacy RC Ralls K Dudash MR Fenster CB Predicting the probability of outbreeding depression Conserv Biol 2011 25 465 475 10.1111/j.1523-1739.2011.01662.x 21486369

[B17] Puurtinen M Mate choice for optimal (K) inbreeding Evolution 2011 65 1501 1505 10.1111/j.1558-5646.2010.01217.x 21521199

[B18] Bateson P Sexual imprinting and optimal outbreeding Nature 1978 273 659 660 10.1038/273659a0 661972

[B19] Bateson P Bateson P Optimal outbreeding Mate Choice 1983 Cambridge: Cambridge University Press 257 277

[B20] Dolgin ES Charlesworth B Baird SE Cutter AD Inbreeding and outbreeding depression in Caenorhabditis nematodes Evolution 2007 61 1339 1352 10.1111/j.1558-5646.2007.00118.x 17542844

[B21] Ryder TB Tori WP Blake JG Loiselle BA Parker PG Mate choice for genetic quality: a test of the heterozygosity and compatibility hypotheses in a lek-breeding bird Behav Ecol 2010 21 203 210 10.1093/beheco/arp176

[B22] Sherman CDH Wapstra E Uller T Olsson M Males with high genetic similarity to females sire more offspring in sperm competition in Peron’s tree frog Litoria peronii Proc Royal Soc B-Biolog Sci 2008 275 971 978 10.1098/rspb.2007.1626 PMC2599940 18230591

[B23] Kokko H Ots I When not to avoid inbreeding Evolution 2006 60 467 475 16637492

[B24] Oh KP Inclusive fitness of ‘kissing cousins’: new evidence of a role for kin selection in the evolution of extra-pair mating in birds Mol Ecol 2011 20 2657 2659 10.1111/j.1365-294X.2011.05118.x 21834140

[B25] Waser PM Austad SN Keane B When should animals tolerate inbreeding Am Nat 1986 128 529 537 10.1086/284585

[B26] Lehmann L Perrin N Inbreeding avoidance through kin recognition: choosy females boost male dispersal Am Nat 2003 162 638 652 10.1086/378823 14618541

[B27] Parker GA Sexual conflict over mating and fertilization: an overview Philos Transac Royal Soc B-Biolog Sci 2006 361 235 259 10.1098/rstb.2005.1785 PMC1569603 16612884

[B28] Thunken T Bakker TCM Baldauf SA Kullmann H Active inbreeding in a cichlid fish and its adaptive significance Curr Biol 2007 17 225 229 10.1016/j.cub.2006.11.053 17276915

[B29] Cockburn A Prevalence of different modes of parental care in birds Proc Royal Soc B-Biolog Sci 2006 273 1375 1383 10.1098/rspb.2005.3458 PMC1560291 16777726

[B30] Lack D Ecological Adaptations for Breeding in Birds 1968 London: Methuen

[B31] Griffith SC Owens IPF Thuman KA Extra pair paternity in birds: a review of interspecific variation and adaptive function Mol Ecol 2002 11 2195 2212 12406233 10.1046/j.1365-294x.2002.01613.x

[B32] Moller AP Ninni P Sperm competition and sexual selection: a meta-analysis of paternity studies of birds Behav Ecol Sociobiol 1998 43 345 358 10.1007/s002650050501

[B33] Kleven O Jacobsen F Robertson RJ Lifjeld JT Extrapair mating between relatives in the barn swallow: a role for kin selection? Biol Lett 2005 1 389 392 10.1098/rsbl.2005.0376 17148214 PMC1626374

[B34] Apanius V Penn D Slev PR Ruff LR Potts WK The nature of selection on the major histocompatibility complex Crit Rev Immunol 1997 17 179 224 10.1615/CritRevImmunol.v17.i2.40 9094452

[B35] Penn DJ Potts WK The evolution of mating preferences and major histocompatibility complex genes Am Nat 1999 153 145 164 10.1086/303166 29578757

[B36] Milinski M The major histocompatibility complex, sexual selection, and mate choice Annual Review of Ecology Evolution and Systematics. Volume 37 2006 Palo Alto: Annual Reviews 159 186 Annual Review of Ecology Evolution and Systematics

[B37] Piertney SB Oliver MK The evolutionary ecology of the major histocompatibility complex Heredity 2006 96 7 21 16094301 10.1038/sj.hdy.6800724

[B38] Landry C Garant D Duchesne P Bernatchez L ‘Good genes as heterozygosity’: the major histocompatibility complex and mate choice in Atlantic salmon (Salmo salar) Proc Royal Soc B-Biolog Sci 2001 268 1279 1285 10.1098/rspb.2001.1659 PMC1088738 11410155

[B39] Reusch TBH Haberli MA Aeschlimann PB Milinski M Female sticklebacks count alleles in a strategy of sexual selection explaining MHC polymorphism Nature 2001 414 300 302 10.1038/35104547 11713527

[B40] Penn DJ The scent of genetic compatibility: Sexual selection and the major histocompatibility complex Ethology 2002 108 1 21 10.1046/j.1439-0310.2002.00768.x

[B41] Penn DJ Damjanovich K Potts WK MHC heterozygosity confers a selective advantage against multiple-strain infections Proc Natl Acad Sci USA 2002 99 11260 11264 10.1073/pnas.162006499 12177415 PMC123244

[B42] Bonneaud C Chastel O Federici P Westerdahl H Sorci G Complex Mhc-based mate choice in a wild passerine Proc Royal Soc B-Biolog Sci 2006 273 1111 1116 10.1098/rspb.2005.3325 PMC1560269 16600889

[B43] Griggio MGM Biard C Penn DJ Hoi H Female house sparrows “count on” male genes: experimental evidence for MHC-dependent mate preference in birds Bmc Evol Biol 2011 11 44 10.1186/1471-2148-11-44 21320306 PMC3044665

[B44] Wagner WE Measuring female mating preferences Anim Behav 1998 55 1029 1042 10.1006/anbe.1997.0635 9632487

[B45] Seutin G White BN Boag PT Preservation of avian blood and tissue samples for DNA analyses Canad J Zool-Revue Canadienne De Zoologie 1991 69 82 90 10.1139/z91-013

[B46] White GC Burnham KP Program MARK: survival estimation from populations of marked animals Bird Study 1999 46 120 139 10.1080/00063659909477239

[B47] Choquet R Lebreton JD Gimenez O Reboulet AM Pradel R U-CARE: Utilities for performing goodness of fit tests and manipulating CApture-REcapture data Ecography 2009 32 1071 1074 10.1111/j.1600-0587.2009.05968.x

[B48] Garnier S Durand P Arnathau C Risterucci AM Esparza-Salas R Cellier-Holzem E Sorci G New polymorphic microsatellite loci in the house sparrow, Passer domesticus Mol Ecol Resour 2009 9 1063 1065 10.1111/j.1755-0998.2009.02552.x 21564839

[B49] Li SH Huang YJ Brown JL Isolation of tetranucleotide microsatellites from the Mexican jay Aphelocoma ultramarina Mol Ecol 1997 6 499 501 10.1046/j.1365-294X.1997.00215.x 9161019

[B50] Richardson DS Jury FL Dawson DA Salgueiro P Komdeur J Burke T Fifty Seychelles warbler (Acrocephalus sechellensis) microsatellite loci polymorphic in Sylviidae species and their cross-species amplification in other passerine birds Mol Ecol 2000 9 2226 2231 11123666 10.1046/j.1365-294x.2000.105338.x

[B51] Griffith SC Stewart IRK Dawson DA Owens IPF Burke T Contrasting levels of extra-pair paternity in mainland and island populations of the house sparrow (Passer domesticus): is there an ‘island effect’? Biol J Linn Soc 1999 68 303 316

[B52] Neumann K Wetton JH Highly polymorphic microsatellites in the house sparrow Passer domesticus Mol Ecol 1996 5 307 309 10.1046/j.1365-294X.1996.00095.x 8673277

[B53] Griffith SC Dawson DA Jensen H Ockendon N Greig C Neumann K Burke T Fourteen polymorphic microsatellite loci characterized in the house sparrow Passer domesticus (Passeridae, Aves) Mol Ecol Notes 2007 7 333 336

[B54] Dawson DA Horsburgh GJ Kupper C Stewart IRK Ball AD Durrant KL Hansson B Bacon I Bird S Klein A Krupa AP Lee J-W Martin-Galvez D Simeoni M Smith G Spurgin LG Burke T New methods to identify conserved microsatellite loci and develop primer sets of high cross-species utility - as demonstrated for birds Mol Ecol Resour 2010 10 475 494 10.1111/j.1755-0998.2009.02775.x 21565047

[B55] Bonneaud C Sorci G Morin V Westerdahl H Zoorob R Wittzell H Diversity of Mhc class I and IIB genes in house sparrows (Passer domesticus) Immunogenetics 2004 55 855 865 10.1007/s00251-004-0648-3 14963619

[B56] Loiseau C Richard M Garnier S Chastel O Julliard R Zoorob R Sorci G Diversifying selection on MHC class I in the house sparrow (Passer domesticus) Mol Ecol 2009 18 1331 1340 10.1111/j.1365-294X.2009.04105.x 19368641

[B57] Loiseau C Zoorob R Robert A Chastel O Julliard R Sorci G Plasmodium relictum infection and MHC diversity in the house sparrow (Passer domesticus) Proc Biol Sci 2011 278 1264 1272 10.1098/rspb.2010.1968 20943698 PMC3049082

[B58] Balakrishnan CN Ekblom R Volker M Westerdahl H Godinez R Kotkiewicz H Burt DW Graves T Griffin DK Warren WC Edwards SV Gene duplication and fragmentation in the zebra finch major histocompatibility complex Bmc Biology 2010 8 29 10.1186/1741-7007-8-29 20359332 PMC2907588

[B59] DeWoody JA Schupp J Kenefic L Busch J Murfitt L Keim P Universal method for producing ROX-labeled size standards suitable for automated genotyping Biotechniques 2004 37 348 15470886 10.2144/04373BM02

[B60] Amos W Wilmer JW Fullard K Burg TM Croxall JP Bloch D Coulson T The influence of parental relatedness on reproductive success Proc Roy Soc Lond B Biol Sci 2001 268 2021 2027 10.1098/rspb.2001.1751 PMC1088844 11571049

[B61] Li CC Weeks DE Chakravarti A Similarity of DNA fingerprints due to chance and relatedness Hum Hered 1993 43 45 52 10.1159/000154113 8514326

[B62] Lynch M Ritland K Estimation of pairwise relatedness with molecular markers Genetics 1999 152 1753 1766 10430599 10.1093/genetics/152.4.1753 PMC1460714

[B63] Van de Casteele T Galbusera P Matthysen E A comparison of microsatellite-based pairwise relatedness estimators Mol Ecol 2001 10 1539 1549 10.1046/j.1365-294X.2001.01288.x 11412374

[B64] Wetton JH Carter RE Parkin DT Walters D Demographic-study of a wild house sparrow population by DNA fingerprinting Nature 1987 327 147 149 10.1038/327147a0 3574474

[B65] Kalinowski ST Taper ML Marshall TC Revising how the computer program CERVUS accommodates genotyping error increases success in paternity assignment Mol Ecol 2007 16 1099 1106 10.1111/j.1365-294X.2007.03089.x 17305863

[B66] Frasier TR STORM: software for testing hypotheses of relatedness and mating patterns Mol Ecol Resour 2008 8 1263 1266 10.1111/j.1755-0998.2008.02358.x 21586016

[B67] Bates D Maechler M Bolker B lme4: linear mixed-effects models using S4 classes. R package version 0.999375-42 2011 http://CRAN.R-project.org/package=lme4

[B68] Burnham KP Anderson DR Model Selection and Multimodel Inference: A Practical Information-Theoretic Approach 2002 2 New York: Springer

[B69] Bolker BM Brooks ME Clark CJ Geange SW Poulsen JR Stevens MHH White JSS Generalized linear mixed models: a practical guide for ecology and evolution Trends Ecol Evol 2009 24 127 135 10.1016/j.tree.2008.10.008 19185386

[B70] Barton K MuMIn: Multi-model inference R Package Version 1.7.2. edition 2012 http://CRAN.R-project.org/package=MuMIn

[B71] Symonds MRE Moussalli A A brief guide to model selection, multimodel inference and model averaging in behavioural ecology using Akaike’s information criterion Behav Ecol Sociobiol 2011 65 13 21 10.1007/s00265-010-1037-6

[B72] Frankham R Do island populations have less genetic variation than mainland populations? Heredity 1997 78 311 327 10.1038/hdy.1997.46 9119706

[B73] Petrie M Krupa A Burke T Peacocks lek with relatives even in the absence of social and environmental cues Nature 1999 401 155 157 10.1038/43651

[B74] Stewart IRK Hanschu RD Burke T Westneat DF Tests of ecological, phenotypic, and genetic correlates of extra-pair paternity in the House Sparrow Condor 2006 108 399 413 10.1650/0010-5422(2006)108[399:TOEPAG]2.0.CO;2

[B75] Veiga JP Boto L Low frequency of extra-pair fertilisations in House Sparrows breeding at high density J Avian Biol 2000 31 237 244 10.1034/j.1600-048X.2000.310215.x

[B76] Cordero PJ Wetton JH Parkin DT Extra-pair paternity and male badge size in the House Sparrow J Avian Biol 1999 30 97 102 10.2307/3677248

[B77] Whitekiller RR Westneat DF Schwagmeyer PL Mock DW Badge size and extra-pair fertilizations in the House Sparrow Condor 2000 102 342 348

[B78] Griffith SC High fidelity on islands: a comparative study of extrapair paternity in passerine birds Behav Ecol 2000 11 265 273 10.1093/beheco/11.3.265

[B79] Charmantier A Blondel J A contrast in extra-pair paternity levels on mainland and island populations of mediterranean blue tits Ethology 2003 109 351 363 10.1046/j.1439-0310.2003.00880.x

[B80] Conrad KF Johnston PV Crossman C Kempenaers B Robertson RJ Wheelwright NT Boag T High levels of extra-pair paternity in an isolated, low-density, island population of tree swallows (Tachycineta bicolor) Mol Ecol 2001 10 1301 1308 10.1046/j.1365-294X.2001.01263.x 11380885

[B81] Fridolfsson AK Gyllensten UB Jakobsson S Microsatellite markers for paternity testing in the willow warbler Phylloscopus trochilus: high frequency of extra-pair young in an island population Hereditas 1997 126 127 132

[B82] Potts WK Manning CJ Wakeland EK Matting patterns in seminatural populations of mice influenced by MHC genotype Nature 1991 352 619 621 10.1038/352619a0 1865924

[B83] Egid K Brown JL The major histocompatibility complex and female mating preferences in mice Anim Behav 1989 38 548 550 10.1016/S0003-3472(89)80051-X

[B84] Olsson M Madsen T Nordby J Wapstra E Ujvari B Wittsell H Major histocompatibility complex and mate choice in sand lizards Proc Roy Soc Lond B Biol Sci 2003 270 S254 S256 10.1098/rsbl.2003.0079 PMC1809963 14667398

[B85] Ekblom R Saether SA Grahn M Fiske P Kalas JA Hoglund J Major histocompatibility complex variation and mate choice in a lekking bird, the great snipe (Gallinago media) Mol Ecol 2004 13 3821 3828 10.1111/j.1365-294X.2004.02361.x 15548294

[B86] von Schantz T Wittzell H Goransson G Grahn M Mate choice, male condition-dependent ornamentation and MHC in the pheasant Hereditas 1997 127 133 140

[B87] Freeman-Gallant CR Meguerdichian M Wheelwright NT Sollecito SV Social pairing and female mating fidelity predicted by restriction fragment length polymorphism similarity at the major histocompatibility complex in a songbird Mol Ecol 2003 12 3077 3083 10.1046/j.1365-294X.2003.01968.x 14629387

[B88] Westerdahl H No evidence of an MHC-based female mating preference in great reed warblers Mol Ecol 2004 13 2465 2470 10.1111/j.1365-294X.2004.02238.x 15245418

[B89] Maitland K Kyes S Williams TN Newbold CI Genetic restriction of Plasmodium falciparum in an area of stable transmission: an example of island evolution? Parasitology 2000 120 335 343 10.1017/S0031182099005612 10811274

[B90] Moro D Lawson MA Hobbs RP Thompson RCA Pathogens of house mice on arid Boullanger Island and subantarctic Macquarie Island, Australia J Wildl Dis 2003 39 762 771 10.7589/0090-3558-39.4.762 14733270

[B91] Lenaghan S Boback S Sundermann C Crim A Hester L Hill B Tedin K Comparison of parasitic infections of Boa constrictor from mainland Belize and the surrounding islands ICOPA XI: Proceedings of the 11th International Congress of Parasitology 2006 Bologna: Medimond S R L 471 474

[B92] Nieberding C Morand S Libois R Michaux JR Parasites and the island syndrome: the colonization of the western Mediterranean islands by Heligmosomoides polygyrus (Dujardin, 1845) J Biogeogr 2006 33 1212 1222 10.1111/j.1365-2699.2006.01503.x

